# Mechanism of Action of Ulipristal Acetate for Emergency Contraception: A Systematic Review

**DOI:** 10.3389/fphar.2015.00315

**Published:** 2016-01-12

**Authors:** Elena Rosato, Manuela Farris, Carlo Bastianelli

**Affiliations:** Department of Gynecological and Obstetrical Sciences and Urological Sciences, “Sapienza" University of RomeRome, Italy

**Keywords:** ulipristal acetate, emergency contraception, ovulation, endometrium, embryo implantation, fallopian tube

## Abstract

Ulipristal acetate (UPA) is now recommended as first choice hormonal emergency contraception (EC), due to its higher efficacy and similar safety compared to Levonorgestrel – EC. Even though all trials demonstrated that the first mechanism of action is inhibition of ovulation, some authors still postulate that a post fertilization effect is also possible, raising the alert on medication and fostering the ethical debate. A Medline database search was performed in order to find recent articles related to UPA’s effects on ovulation, on fallopian tube and on endometrium. We also analyzed the effects on sperm function and pregnancy. All studies conclude that UPA is effective in inhibition of ovulation even when administered shortly before LH peak. The effects on fallopian tube are unclear: according to some authors UPA inhibits ciliar beat through an agonistic effect on progesterone receptors, according to others it antagonizes the progesterone-induced ciliar beat decrease. Concerning the action on endometrium and on embryo implantation most of the studies concluded that low dose UPA used for EC has no significant effect on the decrease of endometrial thickness and on embryo’s attachment, but these results are still matter of debate. Finally recent evidence suggests that UPA modulates human sperm functions while it has no effect on established pregnancy. To date the majority of the evidence concurs in excluding a post-fertilization effect of UPA, even though more studies are needed to clarify its mechanism of action.

## Introduction

To date, three hormonal methods and the use of a copper intrauterine device are available worldwide for emergency contraception (EC), while the use of a fourth hormonal method (low dose mifepristone 10–25 mg) is available only in Armenia, China, Russia, and Vietnam.

First available hormonal EC pills were based on existing combined oral contraceptives preparations, containing ethinyl estradiol (EE) and levonorgestrel (LNG) administered according the so-called Yuzpe regimen (Yuzpe et al., 1974).

Then, it has been shown that the LNG at the dose of 1,5 mg alone was both more effective and better tolerated than the Yuzpe regimen (WHO, 1998).

The World Health Organization (WHO, 1999) pioneered also the trials for using a selective progesterone receptor modulator (SPRM) for EC. These trials led to the development of a second generation SPRM, ulipristal acetate (UPA) at the dose of 30 mg. Use of this SPRM for EC was authorized by the European Medicines since 2009 in Europe (EMEA, 2009) and by the US Food and Drug Administration (FDA) in 2010 and in Asia (US FDA, 2010).

Ulipristal acetate is now recommended as first line treatment for EC, due to its higher efficacy and a similar rate of side effects when compared to LNG-EC.

The exact pharmacological mechanism of action of UPA is still under debate. Even though there is good evidence that the main mechanism of action is inhibition of ovulation, whereas a post-fertilization effect has not been shown, some still consider the latter also possible, raising the alert on medication and fostering the ethical debate because some religious views consider fertilization the initial event of human life.

Aim of this review is to collect experimental and clinical evidence regarding the effects of UPA both before and post fertilization, in order to refine the ethical debate.

This article reviews UPA’s mechanism of action mainly on preventing ovulation, on interfering with oocyte or zygote transport through fallopian tube, and on endometrium receptivity and embryo implantation. The effects of UPA on sperm function and pregnancy were also analyzed (**Table 1**).

**Table 1:** Ulipristal acetate (UPA) effects on ovulation, on the fallopian tube, on the endometrial receptivity and embryo attachment, on sperm function and on pregnancy.

Reference	Type of study	Study sample	UPA dose	Effect studied	Findings/comments
Stratton et al., 2000	Prospective, randomized placebo- controlled study	44 women	10, 50, 100 mg	Effects on ovulation	When administered in mid follicular phase, with a follicle’s diameter of 14–16 mm, a single dose of 10–50–100 mg of UPA causes a dose-dependent delay in the time interval from treatment to follicular rupture and a suppression of estradiol.
Brache et al., 2010	Prospective, double-blind, crossover, randomized, placebo-controlled study	35 women	30 mg	Effects on ovulation	If administered when the size of the leading follicle was ≥18 mm, follicular rupture failed to occur in 59% of cycles. The block/delay ovulation occurred in 100% of women with LH levels very low, and also in 79% of women with LH levels already in the growth phase. Blocking ovulation has not occurred when the LH was at the peak.
Brache et al., 2013	Comparing of three pharmacodynamics studies with similar methodology	163 women	30 mg	Effects on ovulation	At the time of the LH surge, UPA is more effective than LNG and placebo (UPA 79%, LNG 14% and placebo 10%).
Li et al., 2014	*In vitro* experimental study	11 fallopian tubes	0, 20, 200, and 2000 ng/ml	Effects on the fallopian tube	UPA inhibits ciliar beat frequency and muscular contraction of the fallopian tube at the pharmacological dose, probably through an agonistic effect on the tubal progesterone receptor
Yuan et al., 2015	*In vitro* experimental study	26 fallopian tubes	0.1, 1, and 10 μmol/L	Effects on the fallopian tube	UPA dose-dependently antagonized the progesterone-induced ciliar beat frequency decrease
Stratton et al., 2010	Prospective randomized, placebo- controlled study	56 women	10, 50, 100 mg	Effects on the endometrial receptivity	Intake of UPA at different doses in the early luteal phase caused a significant dose-dependent decrease in endometrial thickness: a significant delay in endometrial maturation was seen in the 50 and 100 mg group compared to placebo and the 10 mg group.
Passaro et al., 2003	Prospective randomized, placebo-controlled study	36 women	1, 10, 50, 100, or 200 mg	Effects on the endometrial receptivity	Intake of UPA at different doses in the late luteal phase has no effect on menstrual cycle length for doses from 10 to 100 mg. After 200 mg, all women had early endometrial bleeding.
Williams et al., 2012	2 Phase III randomized double-blind, placebo- controlled clinical trials	546 women	5 or 10 mg taken continuously (13 weeks)	Effects on the endometrial receptivity	After 13 week of UPA treatment, the glandular epithelium appeared inactive, the glandular architecture was altered, and abnormal stromal vessels were also often observed.
Berger et al., 2015	*In vitro* randomized controlled study	20 endometrial constructs	200 ng/ml	Effects on the endometrial receptivity and embryo attachment	There is no significant difference in the attachment of embryos following treatment with UPA compared with controls and no observable degenerative changes in the embryos.
Ko et al., 2014	*In vitro* study	Semen samples with normal semen parameters	0.04, 0.4, 4, and 40 μM	Effects on human sperm function	UPA dose-dependently suppressed progesterone-induced acrosome reaction, hyperactivation and calcium concentration in spermatozoa, by acting as progesterone antagonists.
Levy et al., 2014	Postmarketing pharmacovigilance data collection	1400000 women	30 mg	Effects on pregnancy	UPA seems to have no effect on established pregnancy.

## Pharmacokinetics and Pharmacodynamics

Ulipristal acetate is a derivative of 19-norprogesterone. The half-life after oral intake is 32 h; it binds to plasma proteins for 97–99%, and it is metabolized by the citochrome P450 (Gemzell-Danielsson et al., 2013a).

Ulipristal acetate depending on the location, the presence of coactivators or coinhibitors of gene expression, and the serum levels of progesterone may exert actions of an agonist or antagonist of progesterone.

The binding capacity to glucocorticoid and androgen receptors is lower than its antiprogestin activity: UPA shows *in vivo* antiglucocorticoid and antiandrogen activity only at doses 50-fold higher than those needed for antiprogestin effect (Gemzell-Danielsson and Meng, 2010).

## Effects on Ovulation

In the follicular phase of menstrual cycle the gonadotropins (LH e FSH) levels increase. FSH promotes the differentiation of primary follicle to secondary follicle and acts on the granulosa cells stimulating them to convert testosterone into estradiol (E2), which is released to support the follicle’s growth.

In the pre-ovulatory phase estradiol levels increase and stimulate hypothalamus resulting an increased secretion of LH. In particular estrogens exert a positive feedback stimulating *de novo* progesterone synthesis within the hypothalamus to trigger the LH surge (Micevych and Sinchak, 2011). The LH surge acts on theca cells by stimulating the proteolytic activity resulting in follicular rupture. Ovulation typically occurs 13 to 16 h after the LH peak and 24–26 h after the estradiol peak. The short fertile window typically begins 5 days before, through ovulation, and concludes 1 day after ovulation.

In the initial phases of clinical development (phase I and II) two studies analyzed various pharmacodynamic effects of UPA (kwon as CDB-2914) on ovulation blockade, at different stages of the follicular phase.

When administered in mid follicular phase it has been shown that a single dose of 10–50–100 mg of UPA, with a follicle’s diameter of 14–16 mm, causes a dose-dependent delay in the time interval from treatment to follicular rupture and a suppression of E2. At higher doses, the lead follicle stopped it’s grow and was replaced by a new lead follicle (Stratton et al., 2000).

On the other hand, if administered when the size of the leading follicle was ≥18 mm, follicular rupture failed to occur within 5 to 6 days following treatment in 59% of cycles. The block/delay ovulation occurred in 100% of women with very low LH levels, and in 79% of women with increasing LH levels. The ovulation’s blockade has not occurred in the group of women where LH was at its peak (Brache et al., 2010).

Compared to LNG there are significant differences. An early study (Croxatto et al., 2004) demonstrated that when the follicle reaches 18–20 mm, ovulation is prevented by LNG in only 12% of cycles (compared with 13% in the placebo group). More recently (Brache et al., 2013), it was shown that, at the time of the LH surge, UPA is more effective than LNG and placebo (UPA 79%, LNG 14%, and placebo 10%).

This demonstrates that UPA is effective even when administered shortly before ovulation, when the LH surge has started, a time period when LNG-CE is no longer effective (Jamin, 2015).

The ability of UPA to inhibit ovulation even when it is given just before ovulation is crucial because the probability of conception is the highest (Wilcox et al., 2004) and is due to the particular properties of this SPRM: when progesterone levels are low it acts as an agonist compound, but when they rise, it behaves as an antagonist by blocking the ascent of LH and therefore the ovulatory peak. UPA treatment prevents ovulation presumably by repressing expression of PR-dependent genes critical for ovulation (Nallasamy et al., 2013). When the LH reaches its peak it becomes insensitive to the progesterone’s feedback and to UPA.

This antagonist effect of UPA on PR, has been confirmed by a recent study (Brache et al., 2015) suggesting that initiating desogestrel (DSG) treatment the day after UPA significantly reduced the ovulation delaying effect of UPA.

## Effects on the Fallopian Tube

The fallopian tube plays an important role in human conception. Fertilization normally occurs in the ampullary region of the tube within 24 h after ovulation. Both muscular contractions and cilia activity in the human fallopian tube are involved in the transportation of zygote.

Too rapid or too slow zygote’s tubal transport could cause desynchronization between the zygote itself and the fallopian tube, and/or the blastocyst and the endometrium.

Some studies have suggested that progesterone may suppress ciliary beating frequency (CBF) and muscular contraction in the fallopian tube: cilia beat significantly slower after treatment with high doses of progesterone (Wanggren et al., 2008). Similar results were shown by a recent study indicating that LNG decreases the CBF *in vitro* and *in vivo* (Zhao et al., 2015).

The action of the UPA on the fallopian tube has been investigated in two recent *in vitro* studies.

In the first study (Li et al., 2014), authors demonstrate that UPA inhibits CBF and muscular contraction of the fallopian tube at the pharmacological dose, probably through an agonistic effect on the tubal PR. Based on these results, the authors postulated that UPA:

- exerts an agonistic effect on PR in the fallopian tube on the PR-A isoform, but partial agonist/antagonist activity on the PR-B isoform (Leo and Lin, 2008). It has also been hypothesized, as shown in mouse model, an agonistic action of UPA in the fallopian tube in contrast to the antagonistic action at the ovary (Teilmann et al., 2006).
- up-regulates mRNA expression of the PR (both generic and specific to PR-B).
- down-regulates andrenomedullin, which plays an important role in enhancing the CBF and muscle contraction tone, amplitude, and frequency in fallopian tube (Li et al., 2010).

According to these finding, UPA should increase the risk of ectopic pregnancy, inhibiting tubal function. In contrast to this, no evidence has been shown an increased risk of ectopic pregnancy with the use of UPA for EC (Levy et al., 2014). It should be stressed that the development of an ectopic pregnancy may not be solely explained by delayed embryo transport, but has to be accompanied by an altered tubal environment and/or embryo factors to result in tubal implantation (Shaw et al., 2010).

In contrast Yuan et al. (2015) showed that UPA does not directly affect CBF or the ultrastructure of the cilia, but it dose-dependently antagonized the progesterone-induced CBF decrease. To prove the hypothesis of a progesterone antagonist action the authors highlighted that: Mifepristone, an antagonist of the PR, inhibits progesterone-induced CBF reduction (Mahmood et al., 1998); UPA has been shown to be a progesterone antagonistic without agonistic activity in most studied published (Attardi et al., 2002; Chabbert-Buffet et al., 2005; Xu et al., 2008; Communal et al., 2012; Ko et al., 2014) and finally, their study demonstrates that UPA is able to up-regulate the expression levels of the estrogen receptor (ER) and the PR in the fallopian tubes, while progesterone down regulates them (Horne et al., 2009).

## Effects on Endometrial Receptivity and Embryo Attachment

During the luteal phase, the corpus luteum stimulates the production of progesterone which creates favorable conditions for the implantation of the fertilized egg in the endometrium, consisting in accumulation of glycogen and lipid granules in the cytoplasm of stromal cells (decidual transformation).

While the effects of UPA on ovulation are well recognized, the controversy arises on its action on endometrium receptivity, in contrast with LNG, on which there is a unanimous opinion in believing that it has no effect on implantation (Lalitkumar et al., 2007; Meng et al., 2009; Palomino et al., 2010).

When in her pre-ovulatory study Brache reported that the effects of UPA at the LH peak are null (Brache et al., 2010), some authors (Mozzanega et al., 2013) pointed out that, if UPA has no effect on the LH peak, it should not have any effects two days before ovulation, in contradiction with the conclusive statement of the paper and therefore the very high UPA’s effectiveness in preventing pregnancies which does not decrease with the time elapsed from unprotected intercourse (Fine et al., 2010; Glasier et al., 2010) should be explained by a post-fertilization effect.

There are two major early clinical phase studies on the postovulatory effects of UPA.

In the first (Stratton et al., 2010), the authors comparing early luteal phase treatment with placebo, 10, 50, 100 mg unmicronized UPA showed that UPA caused a significant dose-dependent decrease in endometrial thickness: a significant delay in endometrial maturation was seen in the 50 and 100 mg group compared to placebo and the 10 mg group upon biopsy four to 6 days after ovulation.

On the basis of these results, (Gemzell-Danielsson et al., 2013a,b, 2014) in several subsequent reviews concludes that low dose UPA (30 mg) as used for EC has no significant effect on endometrium.

Some authors (Glasier et al., 2010; Hillemanns and Hepp, 2013; Mozzanega et al., 2013) confuted that the 50 mg UPA tablet used in the early luteal phase study involves a significant reduction in endometrial thickness, and it is pharmacokinetically identical to the 30 mg micronized formulation used in the EC.

The second luteal phase study (Passaro et al., 2003) shows that intake of UPA at different doses in the late luteal phase has no effect on menstrual cycle length for doses from 10 to 100 mg.

More recently, a study of the endometrial morphology with UPA taken continuously (13 weeks) at lower doses (5 or 10 mg), was carried out in a group of patients with uterine myomas (Williams et al., 2012). Following treatment, the glandular epithelium appeared inactive, the glandular architecture was altered and abnormal stromal vessels were also often observed (the so called PRM-associated endometrial changes PAEC).

In a similar study (Donnez et al., 2012), at 13 weeks of treatment with different doses of UPA the mean endometrial thicknesses was 9.4 mm in the group receiving 5 mg and 10.7 mm in the group receiving 10 mg. The PAEC were observed in the same percentage of women receiving 5 mg or 10 mg UPA.

Finally, the effect of UPA on human implantation has been extensively studied utilizing an *in vitro* three-dimensional endometrial model. With this *in vitro* model, it was shown (Berger et al., 2015) that there is no significant difference in the attachment of embryos following treatment with UPA compared with controls and no observable degenerative changes in the embryos. Moreover it was found that the level of genes of several factors believed to be vital for embryo implantation remain unaltered in the endometrial construct after exposure to UPA.

## Effects on Human Sperm Function

In their transit in female reproductive tract, spermatozoa are exposed to increased levels of progesterone secreted by the cumulus cells and the corpus luteum. Progesterone in human follicular fluid is essential for acrosome reaction induction, permitting calcium influx into spermatozoa.

Initially (Munuce et al., 2012) it was reported that UPA does not modify sperm capacitation, nor induce acrosome reaction (no agonist effect on PR) or prevent human follicular fluid induced acrosomal reaction (no antagonist effect on PR).

A more recent study (Ko et al., 2014) has shown that UPA dose-dependently suppressed progesterone-induced acrosome reaction, hyperactivation, and calcium concentration in spermatozoa, concluding that UPA modulate human sperm functions by acting as progesterone antagonists.

In contrast to UPA, *in vitro* (Brito et al., 2005) and *in vivo* (Do Nascimento et al., 2007) studies found that LNG at doses used for EC has no direct effect on sperm function.

## Effects on Pregnancy

In a post marketing experience on more than 1 million women (Levy et al., 2014) 376 pregnancies have been observed. Of the 232 with a known outcome, 28 ended with live births, 34 were miscarriages, 151 induced abortions, 4 ectopic pregnancies, and 15 which are still ongoing.

The observed rate of miscarriages and ectopic pregnancy were not increased when compared with the rate observed in the general population.

## Discussion and Conclusion

Several studies provide strong direct evidence that both UPA and LNG act by preventing or delaying ovulation. The difference between the effects of UPA and LNG on ovulation is in their window of action. When the follicular size is >18 mm, LNG is unable to prevent ovulation while UPA acts until before the LH peak immediately before ovulation (Brache et al., 2013).

Those that advocate a post fertilization effect of UPA stress that its effectiveness in preventing pregnancies is very high, although it is taken in proximity of ovulation. On this regard, some key points need to be considered.

Firstly, the published articles on the postovulatory effects UPA rely on already published studies and not on new *in vitro/vivo* studies. Moreover in many of these debates, there is not a clear distinction between the time of ovulation and the time of sexual intercourse. The “5 days after” refers to sexual intercourse event and not to the time of ovulation. This could be considered as the same kind of miswording which referred to LNG-EC as “the day after” pill. On this subject, some authors (Hillemanns and Hepp, 2013) sustain that when unprotected intercourse occurs for example 12 h after ovulation and UPA is taken “5 days later,” then UPA must have other effects than delaying ovulation.

Secondly, in studies on UPA effects on the fallopian tube (Li et al., 2014; Yuan et al., 2015) is unclear how it affects fertilization and/or transport of the fertilized egg and therefore the embryo implantation.

Thirdly, the authors that showed an endometrial suppression after treatment with the same SPRM utilized for treatment of myomas (Williams et al., 2012) did not demonstrate that this is also true for the UPA dosage utilized for EC.

Finally, the abortifacient role of Mifepristone (“RU-486”), at higher doses, could influence negatively the attitude toward same family compounds. The drug is approved for use in combination with misoprostol in a number of countries to terminate a pregnancy, given at the dose of 200–600 mg which is 8- to 60-fold greater than the dose used for EC (WHO, 2012).

On the other hand, it should be pointed out that the exact mechanism of action of UPA is not completely clarified. In particular it is still poorly understood when it acts as agonist or antagonist of progesterone. Knowledge of its precise mechanism of action would be helpful to better understand its effects on target organs.

If there would be a method of identifying fertilization, it could have been the only way to show if UPA acts in the early phase of the zygote, but attempts to identify a molecule that could operate as a early marker of fertilization have not be successful. In particular a protein called the “early pregnancy factor” (EPF) was first described in mice as an immunosuppressive agent (Morton et al., 1974) and subsequently identified in additional species and humans (Morton et al., 1982; Tinneberg et al., 1984). The biological features of EPF suggest an opportunity for early pregnancy diagnosis: it could be, in the future, a marker to verify UPA’s mechanism of action (Hatzel et al., 2015).

The ethical problem arises in considering the possibility of its putative post fertilization effect, based on the assertion that it causes abortion.

By definition, contraception comprises all methods capable of preventing pregnancy as defined by the WHO. Accepting this definition, hormonal EC includes all methods acting after intercourse, but before implantation. To date, the mechanism of action appears rather related to postponing ovulation, having an effect on sperm function while no effect on endometrium has been shown at clinically relevant doses.

## Author Contributions

ER and MF wrote the articles, CB supervised the work.

## Conflict of Interest Statement

The authors declare that the research was conducted in the absence of any commercial or financial relationships that could be construed as a potential conflict of interest.
